# Social structures, power and resistance in monist sociology: (New) materialist insights

**DOI:** 10.1177/1440783317730615

**Published:** 2017-09-11

**Authors:** Nick J Fox, Pam Alldred

**Affiliations:** University of Sheffield, UK; Brunel University London, UK

**Keywords:** monism, new materialism, ontology, posthumanism, power, resistance, social structure

## Abstract

Though mainstream sociological theory has been founded within dualisms such as structure/agency, nature/culture, and mind/matter, a thread within sociology dating back to Spencer and Tarde favoured a monist ontology that cut across such dualistic categories. This thread has been reinvigorated by recent developments in social theory, including the new materialisms, posthumanism and affect theories. Here we assess what a monist or ‘flat’ ontology means for sociological understanding of key concepts such as structures and systems, power and resistance. We examine two monistic sociologies: Bruno Latour’s ‘sociology of associations’ and DeLanda’s ontology of assemblages. Understandings of social processes in terms of structures, systems or mechanisms are replaced with a focus upon the micropolitics of events and interactions. Power is a flux of forces or ‘affects’ fully immanent within events, while resistance is similarly an affective flow in events producing micropolitical effects contrary to power or control.

Sociology has been frequently keen to expose the binary oppositions or ‘dualisms’ that invest much human thinking: systems of thought that have been used culturally to differentiate and divide human from animal (Peggs, 2012: 2), man from woman (Braidotti, 2011: 39), noble from commoner, ‘modern’ from ‘traditional’ cultures (Bhambra, 2016; Spivak, 1988), straight from queer (Weeks, 2012), saint from sinner (Durkheim, 2005), normal from pathological (Foucault, 1981: 44), purity from pollution (Douglas, 1984). Dualisms work by privileging one pole of a binary opposition at the expense of another (Derrida, 1976), and serve typically to assert power and privilege of one class, gender, sexuality, race and so on over others. In so doing, they establish the premises and cognitive armoury for patriarchy, colonialism, homophobia and class or caste systems, the scapegoating of ‘foreigners’ and the anthropocentrism that underpins activities from industrialised farming to global environmental policy (Fox and Alldred, 2017).

While sociological analysis has exposed the dualist schemata used in daily life, it has not been immune to the seductions of binary oppositions itself. Sociological dualism was manifest in Marx’s dichotomy of labour/capital and Durkheim’s distinction between traditional and modern societies, but most pervasively in the dualism of agency/structure and a nature/culture divide that has arguably underpinned the disciplinary development and professional closure of sociology itself (Benton, 1991; Meloni, 2016). Many of these sociological binaries have been the subject of fierce debate within the discipline (Karakayali, 2015). The social sciences have occasionally been strongly criticised for sustaining contemporary dualisms, for example, anthropology’s early collusion with racist and colonialist theories (Gravlee and Sweet, 2008: 28) or second-wave feminist essentialism (Braidotti, 2011: 129; New, 1998: 349). We shall not attempt however to document the long history of dissent, commentary and criticism around the sociological dualisms of agency/structure (Gleeson and Knights, 2006; Knights and Willmott, 1983; Mouzelis, 2014; Piiroinen 2014) or around the distinctions between nature/culture, human/non-human, animate/inanimate (Benton, 1991; Stevens, 2012; Walker, 2005). Figure 1 summarises some of the most common binary oppositions to be found within sociological discourses.

**Figure 1.**
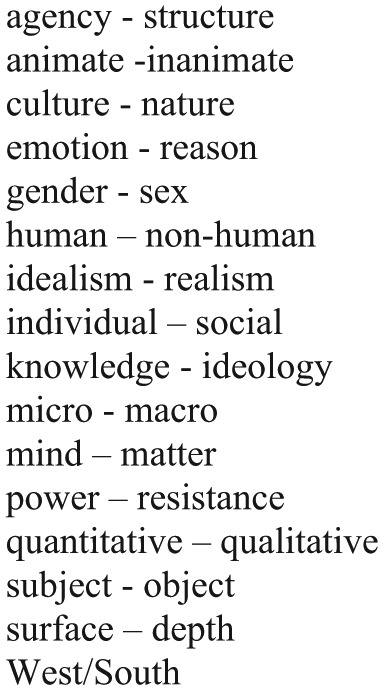
Sociological binaries

Sociology’s self-positioning in relation to these binaries made it the target for post-structuralist theorists, who ruthlessly deconstructed the oppositions. By privileging culture over nature (for example, by emphasising gender – a cultural formation – at the expense of biological sex) sociology established the credentials of the social world, which is of course, the discipline’s chosen subject-matter (Game, 1991: 33, Meloni, 2016). The opposing elements of the agency/structure binary – endlessly re-worked in structuralist, interactionist, historical materialist, structuration and realist theories (DeLanda, 2006: 9-10) – has been criticised for generating two contrary tendencies within sociology. On one hand, structuralist sociologies’ concern with the determining features of social norms, roles, rituals and systems (for instance, Marx’s focus upon an economic ‘base’ structuring social interactions or critical realism’s commitments to uncovering underlying ‘mechanisms’), overemphasise social continuities and stability (Wrong, 1961) at the expense of flux and possibility. On the other, an emphasis upon human agency has led to an ‘undersocialised’ sociology that privileges reason and reflexivity, desires and emotions, while downplaying the social and material contexts of events/interactions (Shilling, 1997).

This critique of sociological dualism poses the interesting question of what sociology might look like were it to eschew entirely such binary oppositions. The need to address this question arises as a consequence of the recent engagements between the social sciences and new materialist perspectives such as actor-network theory, non-representational theory, feminist posthumanism, assemblage theories and Spinozist theories of affect (Braidotti, 2013; Coole and Frost, 2010; Fox and Alldred, 2017).^1^ These, it has been argued, cut across or are ‘transversal to’ many of the binaries in Figure 1, including mind/matter, nature/culture, structure/agency, micro/macro and surface/depth (Fox and Alldred, 2017; Karakayali, 2015; van der Tuin and Dolphijn, 2010: 153).

The choreography of the article is as follows. We begin by exploring how social theory has been ‘flattened’ (Karakayali, 2015: 742) in the monist and materialist sociological manifestos of Bruno Latour (2005) and Manuel DeLanda (2006). We then apply this flattened ontology to re-think the key sociological concept of ‘social structure’, which along with its binary opposite ‘agency’ is effectively dissolved by this transversal move. Finally, we assess the consequences for understanding power and resistance, terms that in conventional sociology have been frequently predicated upon an agency/structure duality.

## Monism, materialism and sociology

Within contemporary sociology, monist ontologies are demonstrated floridly within the posthuman and ‘new’ materialisms that have emerged within the social sciences and humanities. Whereas historical materialism focused on the development of social institutions and practices within a broad economic and political context of material production and consumption (Edwards, 2010: 282), the materiality addressed by these new materialisms is plural, open, complex, uneven and contingent, and should be understood ‘in a relational, emergent sense’ (Coole and Frost, 2010: 29) that draws together natural and social worlds (2010: 20). These positions (which for conciseness, we henceforth refer to as ‘materialist’) have in common a commitment to immanence (Deleuze, 1988: 124); in other words, ‘a philosophy of becoming in which the universe is not dependent upon a higher power’ (Connolly, 2011: 178) – powers that might include God, fate, evolution, life-force, *Gaia*, mechanisms, systems or structures. Instead we are to explore events and interactions within a ‘plane of immanence’ that possesses ‘no supplementary dimension’ (Deleuze, 1988: 128).

The monism of these materialisms is revealed in three ontological moves. First, they cast to one side a foundational boundary dispute between ‘social’ and ‘natural’ sciences (Meloni, 2016), questioning the very separation between nature and culture (Latour, 2005: 13). Instead, they link the production of the world and everything ‘social’ and ‘natural’ within it to a wide variety of forces, from physical interactions, to biological processes, to social encounters, through to thoughts, desires, feelings and memories (Braidotti, 2000: 159; DeLanda, 2006; 5).

Second, they regard the material world and its contents not as fixed, stable entities, but as relational, uneven and in constant flux (Barad, 2007, Coole and Frost, 2010: 29; Lemke, 2015), consequent entirely upon the micropolitical forces deriving from matter’s interactions within events. For Deleuze, human bodies and all other material, social and abstract entities have no ontological status or integrity independent of that produced through their relationship to other similarly contingent and ephemeral bodies, things and ideas (Deleuze, 1988: 123). ‘Assemblages’ of relations develop in unpredictable ways around actions and events (Deleuze and Guattari, 1988: 88), ‘in a kind of chaotic network of habitual and non-habitual connections, always in flux, always reassembling in different ways’ (Potts, 2004: 19).

Third, the relationality of the world is operationalised via an understanding of agency that no longer privileges human action. Rather, all matter is ‘affective’ – it possesses a ‘capacity to affect and be affected’ (Deleuze and Guattari, 1988: 127–8), whether it is human or non-human, animate or inanimate (DeLanda, 2006: 4; Mulcahy, 2012: 10; Youdell and Armstrong, 2011: 145). Replacing (human) agency with ‘affect’ serves as an ethical and political counter to the humanism of the social sciences, supplying the basis both for an anti-humanist critique of the destructive capacities of humans in the Anthropocene (Lovelock, 2007: 141) and to reintegrate humans within ‘the environment’ (Fox and Alldred, 2016), thus underpinning a more positive posthumanism (Braidotti, 2006: 37). The latter, according to Braidotti, can be a basis for an eco-philosophy that establishes a continuum between human and non-human matter (Braidotti, 2006: 41, 2013: 104).

When applied to sociology, these aspects of contemporary materialism’s monism (van der Tuin and Dolphijn, 2010: 155) or ‘flat ontology’ (DeLanda, 2005: 51) collapse or cut across a range of conventional social theory dualisms – including agency/structure, nature/culture, animate/inanimate, micro/macro, reason/emotion, surface/depth, word/world and mind/matter (Braidotti, 2013: 4–5; Coole and Frost, 2010: 26–7; Deleuze and Guattari, 1988: 23; van der Tuin and Dolphijn, 2010: 157).^2^ The elision of nature/culture and human/non-human dualisms has been addressed elsewhere (Barad, 2007; Braidotti, 2013; Fox and Alldred, 2016; Haraway, 1991; Karakayali, 2015: 741–2), and we address here another critical issue for sociology: the dissolution by sociological monism of any conception of ‘social structure’, and the knock-on consequences for two other key sociological concepts: power and resistance. As a starting position for this endeavour, we briefly explore the sociological working out of an immanent social world *sans* structures, systems or ‘underlying mechanisms’, in the manifestos for a materialist sociology of Bruno Latour (2005) and Manuel DeLanda (2006).

Latour’s (2005) *Re-assembling the Social* develops ideas from actor-network theory (ANT) to establish his agenda for a ‘sociology of association’ (Latour, 2005: 9). ANT is a well-established sociological perspective that acknowledges non-human agents (often referenced by the French term ‘*actants*’) as contributors to social production within transient relational networks (Latour, 1996: 370; Law, 1999: 4) that encompass both ‘social’ and ‘natural’ elements (Law, 1992: 379). Latour uses this heterogeneity of social production to offer a concerted critique of the sociological understanding of ‘the social’ as a distinct domain of reality (Latour, 2005: 4). His contrary view is that ‘the social’ is not a realm distinct from other materialities such as biology or physics.

The task of the sociologist is consequently not to describe and explain ‘social forces’, but to explain how a range of heterogeneous elements from the physical, biological, economic, semiotic and other ‘realms’ may be assembled to produce this or that social aggregation (2005: 5–6). These aggregations (such as a nation, a corporation, a social institution, a social category or an aspect of human culture) are the outcomes, not the causes of interactions. Sociology should not restrict itself to studying social ties, but instead ‘travel wherever new heterogeneous associations are made’ (2005: 8), in order to understand how the social is continually assembled from non-social associations.

Latour (2005: 8) targets ‘critical sociology’ – which we take to mean approaches such as critical realism and Marxism – that have sought to explain the social in terms of ‘deep’ or underlying structures or mechanisms. Latour’s monistic sociology rejects any sense of social forces or structures working ‘behind the scenes’, replacing these entirely with localised, short-lived interactions or associations (2005: 65–6) that constitute what is commonly called ‘the social’. Such structural ‘explanations’ epitomise a sociology that proffers explanatory concepts such as ‘patriarchy’ or ‘neoliberalism’, concepts that – in his view – themselves need to be explained (2005: 130–1).

The work of Manuel DeLanda applies a Deleuzian/Spinozist toolkit (Deleuze, 1988) of relationality, assemblages and affects to establish his materialist sociology. In *A New Philosophy of Society*, he argues against the ‘organic’ models of society that have shaped sociology from Parsonian functionalism to Giddens’s structuration theory (DeLanda, 2006: 8–9). These sociologies are based on a ‘superficial analogy between society and the human body’ (2006: 8), and depend upon ‘relations of interiority’ (2006: 9), meaning that component elements (the ‘organs’) have inherent attributes or properties that are manifested only when constituted with other specific elements within a whole (the ‘organism’). So, for example, ‘teachers’ and ‘students’ (the parts) manifest their particular properties when interacting together as elements within a school or college (the whole).

Instead, DeLanda replaces the ‘organism’ with the ‘assemblage’ (DeLanda, 2006: 9–10) to establish a model of collectivities whose emergent properties derive entirely from ‘relations of exteriority’ (2006: 10–11; see also Buchanan, 2000: 120). Here, a relation such as a human body or a non-human object may be detached from one assemblage and plugged into another, within which it will have differing interactions and consequently exercise different capacities. So a relation may become a ‘learning-body’ when it is part of an assemblage in which it interacts with ‘teaching-bodies’ and ‘knowledge’; these relational capacities, in turn, establish the assemblage’s capabilities to serve as ‘school’ or ‘college’. But detached from this assemblage and plugged in elsewhere, the former ‘learning-body’ may manifest different capacities (for instance, as a ‘worker’ or a ‘lover’) as it interacts with other bodies in a ‘workplace-assemblage’ or a ‘sexual relationship-assemblage’, respectively.

DeLanda uses this Deleuzian analysis of relations and capacities as the foundation for an immanent sociology that can yet analyse social production at multiple societal levels. In place of a ‘deep level’ of social structures or underlying social mechanisms that provide conventional sociology with its explanations of phenomena, he offers a flat (DeLanda, 2005: 51) ontological ‘layering’ of assemblages from micro to macro; from interpersonal interactions such as a conversation (DeLanda, 2006: 53-55) to social organisation at the level of the state (2006: 113–16). Every social entity – for instance, an industrial corporation – emerges from interactions occurring at a smaller scale, such as a network of managers, suppliers and distributors (2006: 75). However, at each level, entities retain a degree of autonomy, enabling social investigations to be undertaken while avoiding both micro- and macro-reductionism (2006: 119).

Latour’s and DeLanda’s statements give a flavour of how contemporary materialist scholarship can inform and indeed re-make sociology. DeLanda’s work supplies an ontology of relationality, which reverses the conventional hierarchy, in which an entity’s relations are subordinate to the entity’s essence (Buchanan, 2000: 120); in this ontology an assemblage is not to be treated as an essence in its own right (DeLanda, 2006: 4), nor does it exert force over its assembled relations. Rather, what relations can do within an assemblage depends entirely upon the forces or ‘affects’ that relations exert upon each other (Deleuze, 1988: 101). Meanwhile, Latour’s (2005: 24) admonition to resist ‘structural’ explanations suggests a starting-point from which to explore empirically the interactions of natural and social relations in events. We draw these two perspectives together in the following section.

## Social production beyond structure or system

Latour (2005: 7) has argued that structural or systemic explanations are frequently invoked to make sense of perceived patterns or replications of particular social formations, often in relation to social divisions, inequality or social disadvantage, and to explain constraints or limits on human action or outright oppression. These sociological ‘explanations’ include ‘capitalism’, ‘racism’, ‘patriarchy’, ‘neoliberalism’, ‘the state’, ‘science’, ‘religion’ and so on, phenomena which – in Latour’s view – are precisely the things that themselves require explaining (2005: 8). This assessment flies in the face of much received sociological wisdom, in which models of social structure, social systems and social mechanisms have been applied conceptually, from historical materialism to systems theories to critical realism. In Latour’s ontology ‘there exists nothing behind those activities, even though they might be linked in a way that does produce a society – or doesn’t produce one’ (Latour, 2005: 8).

Ruling out any recourse to overarching ‘social structures’ or ‘systems’ or underlying ‘mechanisms’ as explanations of continuity and change means that the task of sociological inquiry is no longer to reveal the hidden social forces at work in law, science, religion, organisations or elsewhere. A materialist sociology must consequently analyse forces and social relations, power and resistance from within the immanent, relational micropolitics of events, activities and interactions themselves. Later in this article we explore what this flattened ontology means for understanding manifestations of ‘power’ and ‘resistance’. First, we assess the flat, immanent landscape of a sociology beyond structure or system, using ‘the market’ as an illustration.

Sociologists have variously theorised the capitalist ‘market’ as a social structure (Swedberg, 1994), as embodying structural relations of governance, law and property rights (Fligstein, 1996), or as ‘embedded’ within structural social relations (Granovetter, 1985). In all these various perspectives, the market structures or systematises the social relations of actors in a capitalist society; the structural character of ‘the market’ has then been used as a sociological explanation for other social processes, such as shifts in how education and health care are delivered in contemporary capitalist societies (Hermann, 2010; Lipman, 2011). Our concern, however, is not with which of these concepts – structure, system or mechanism – might best be invoked to supply an explanation of the workings of the market. From the materialist perspective established earlier, each of these rival ‘explanations’ rests upon a binary model of society in which ‘human agency’ is pitted against a distinct realm of social formation (sometimes described as a ‘base’ or a ‘deep level’, and sometimes – as in Giddens’s (1981: 27) structuration theory – simply as a ‘medium’) that in some way shapes, constrains or on occasions facilitates action.

A non-binary reconceptualisation of ‘the market’ necessarily starts from a very different place, by looking not at structures but at ‘market-events’, in other words concrete manifestations of markets and the activities that take place within them. We can begin this re-think with the Deleuzian conceptual toolkit outlined earlier: relations, assemblages and affects. At its simplest, a market-event could be summarised as an assemblage comprising

commodity – individual A – individual B – money

The interactions between these relations will derive entirely from the affects (capacities to affect and be affected) between them. Deleuze and Guattari (1988: 453) noted the distinctive character of the capitalist relation that enables a commodity to be traded by A in return for a mutually agreed sum of money from B, according to ‘market principles’. What is remarkable, they argued, is not the presence of overarching structures or underlying mechanisms that assure this exchange, but rather the exceptionally de-contextualised or ‘de-territorialised’ capacity of this relation to occur, unconstrained by contextual factors such as the relative statuses of A and B, which would preclude open transactions in feudal social forms.^3^

Markets, DeLanda (2005: 17) argues, should be seen first and foremost as material places that are assemblages of humans and the things, services or abstractions they trade. The development of a ‘market economy’, he goes on, emerges from the geographical interactions of these discrete marketplaces, which across time and space facilitate national and international trading (2005: 18). To this we might add the material affects that derive from repeated, routinised and habituated pattern of interactions, memories, experiences and outcomes that encourage marketised behaviours. It is out of these multiple disparate and often divergent events that what appears to be a stable market structure or system (and indeed ‘capitalism’ itself) emerges.

However, such market behaviours and orientations possess far less stability within a non-binary conceptualisation than in traditional sociological theories. From such a perspective, it is solely the various affects within individual events that promote or constrain a ‘market relation’, and the latter is continually challenged by new relations and affects that may de-stabilise commercial interactions, impose constraints on markets and introduce different models of social interaction such as collectivism or state intervention. This instability and flux, we would argue, reflects far better what actually goes on in economic and social transactions than claims of market hegemony (see for example, Berry, 2014).

Consequently, something (the market) that has been used on occasion as a structural ‘explanation’ in sociological studies – for instance to claim the hegemony of contemporary society’s neoliberal orientation (Jessop, 2002: 455) – may be re-thought in terms of a series of material and relational events or assemblages, in which intra-actions are continuously produced and reproduced. ‘Macro’ relations such as government policy, or the cultural and social processes described in Bourdieu’s (2005) essay on the market, can be incorporated indirectly into the ‘market-assemblage’ in terms of the affectivity of those policy initiatives as they influence human and non-human relations. A similar approach that focuses on events may be used to re-think other ‘explanations’, such as patriarchy or consumerism, making these the things that themselves need to be explained, rather than positing them as structural, systemic or mechanical explanations. What has appeared structural or systemic to sociologists is rather a product of reproduced affect economies or intra-actions between assembled relations. This conclusion establishes the materialist framework from which to explore movements of power and resistance within assemblages.

## The relationality of power/resistance

Power and resistance are concepts that have been foundational both to theorising social change and to practical interventions to address injustices or inequalities through practice, policy or activism (Boudon, 1991; Dale and Kalob, 2006; Fox and Alldred, 2017). Though power has been variously conceptualised in social theory, sociologists have been wary of ontologies that reduce power to human decision-making, regarding such models as ‘one-dimensional’ (Lukes, 2004: 19), or failing to acknowledge the duality of agency/structure (Giddens, 1981: 49–50). Dualist approaches to power include Marx’s analysis of the consequences of the social and economic relations of capitalist production (Gramsci 1971: 181–2; Nigam, 1996: 8–9), Connell’s (1987) analysis of gender and social structure, and Parsons’ (1963: 232) assessment of power as a circulating medium that enables a complex society to work effectively and manage resistance.

The de-privileging of human agency and its rejection of ‘another level’ of structures or mechanisms together problematise notions of power theorised as top-down structural forces, as an aspect of structuration (Giddens, 1981: 49), or as an amorphous ‘stuff’ that permeates the everyday social world and social interactions. We can enunciate the precise challenges that a flat ontology poses for theories of power. First, within such an ontology, phenomena described by sociologists as ‘power’ may comprise nothing more nor less than the interactions between assembled relations as they affect and are affected (Braidotti, 2013: 188–9; Patton, 2000: 52). Power is consequently *integral* to what goes on in this daily round of events; to be treated not as a unitary force upon citizens, but revealed and deployed at the very local level of actions and events (Barad, 2001: 94).^4^ Thus, for example, the gendered expressions of power and oppression between young people in school settings are not products of abstracted structural forces such as ‘patriarchy’ or ‘hegemonic masculinity’. Instead, they are the outcomes of micropolitical material forces and intensities operating within the daily round of events in and out of the classroom (Alldred and Fox, 2015).

Second, within a monist sociology, power is necessarily transient and fluctuating – a momentary exercise by one relation over another. The apparent regularities or continuities in power discerned by sociologists (for instance, patriarchal power of one gender over another, or the dominance of market models of social interaction in contemporary society) will depend upon continued replication of these specific forces or affects between assembled relations, thereby sustaining particular assemblage micropolitics. These micropolitical patternings in time and space may lead to continuities of hierarchic relations, to produce the semblance of overarching structures or systems or underlying mechanisms (for instance, ‘patriarchy’ or ‘capitalism’). However, this regularity is illusory: power can have continuity only so long as it is replicated in the next event, and the one after that, and may quickly evaporate when affects in an assemblage alter.

From this analysis, it follows that a materialist understanding of power (and of resistance to power) will be radically empirical, to be both understood and researched locally and micropolitically, focusing upon the affects between both human and non-human relational materialities within events, actions and interactions (assemblages). What then of resistance? Sociologists have always recognised an intimate association between power and resistance – where there is one, there is also the other, almost by definition (Lupton, 1997: 102). Often this opposition of power and resistance has been underpinned by structure/agency dualism (DeLanda, 2006: 10), with resistance conceptualised as the response of a plucky human agent unwilling to be ground down by the coercive powers of social structures, a bureaucratic iron cage (Weber, 1930: 181) or the daily grind of work (Marx and Engels, 1952: 52).

Once again, a materialist and relational ontology developed earlier requires that resistance is conceived in terms of an assemblage micropolitics founded upon relations of exteriority. This shifts the basis for resisting powerful forces away from an essentialised human agent with fixed attributes, and towards the relational capacities of assembled bodies, things and social formations within assemblages. What has conventionally been termed ‘resistance’ is a flux of forces or affects in an assemblage that produces micropolitical effects contrary to power or control (Deleuze and Guattari, 1988: 216), whether as organised or more haphazard and random resistance – moments as well as movements.

Elsewhere (Fox and Alldred, 2015) we have described two micropolitical movements within assemblages that produce the capacities of bodies and other relations. The first of these we termed specification/generalisation (based on Deleuze and Guattari’s [1988: 88–9] movements of territorialisation/de-territorialisation), which describe how a body’s or other relation’s capacities are either focused or turned loose by the affects in an assemblage. The development of social identities (as ‘male’, ‘disabled’, ‘heterosexual’ and so forth) is a good example of a specification: identities that may subsequently be generalised by other social or cultural affects. The second micropolitical movement we have called aggregation/disaggregation (a recasting of Deleuze and Guattari’s [1984: 286–8] distinction between ‘molar’ and ‘molecular’ forces). This differentiates between forces/affects that classify or group relations together, and those that single them out as unique. So, for instance, classifying (aggregating) bodies into social classes, races or genders has the effect of lumping together quite disparate persons (Colebrook, 2013: 36), whereas mentoring or sponsoring may bring out a person’s unique capacities.

It would be simplistic, however, to link ‘power’ with specification and aggregation, and ‘resistance’ with movements of generalisation and disaggregation (cf. Patton, 2000: 65–6). Though the former are frequently the means whereby relations in assemblages assert control and thus power over other relations, we cannot assume that resistance is always associated with generalisation and singularity. As noted earlier, a capitalist marketplace is actually a radically unconstrained space, in which anyone can trade with anyone (Deleuze and Guattari, 1984: 222). Resisting the forces of the free market in such circumstances may actually entail individual consumers aggregating together and re-specifying themselves as a ‘workers’ collective’ that refuses to accept the anarchy of the marketplace.

It may therefore be more accurate to see power and resistance as dual fluxes that permeate all assemblages, a shifting balance that is never finally settled. Defining a certain affect as an assertion of power or an effort at resistance is less important than assessing the capacities that these affects produce. Rather than presenting certain events as examples of coercive or disciplinary power, and others as instances of resistance, what may be important is to document how transient assemblages are stabilised, what material forces enable certain relations to consistently specify (territorialise) others, and how bodies are forced to resist in more and more obscure and desperate ways. Furthermore, the fluctuating character of assemblage micropolitics means that ‘power’ and ‘resistance’ wax and wane, shift and reverse continually: all events are consequently sites in which both ‘power’ and ‘resistance’ may be discerned. This analysis also unsettles a simplistic equation of power with action and resistance with reaction.

## Discussion

A growing number of social scientists (for a review, see Fox and Alldred, 2015) are embracing opportunities offered by contemporary materialist and posthuman ontologies to establish approaches to social theory and research not trammelled by humanism and essentialism, or that cut across dualisms between human and non-human, nature and culture, micro and macro, mind and matter (DeLanda, 2006: 26, 46; van der Tuin and Dolphijn, 210: 156). In this article we have disclosed and sought to work through some of the implications of monist ontology for key sociological concepts such as agency, structure, power and resistance. We have suggested that abolishing some cherished binaries does not make the sociological sky fall in. While concepts like social structure, the critical realist pursuit of underlying mechanisms (Danermark et al., 2002: 59), and overarching notions such as ‘neoliberalism’ and ‘patriarchy’ all have to be abandoned, and power and resistance have to be reconceptualised as operating locally and in ways that are far more fragmentary, monist ontology still enables the pursuit of a sociological project, both theoretically and empirically.

What then might be the up side of this monism (that is also a pluralism) for a sociological imagination? We would argue that there are three main opportunities. First, dissolving sociological dualisms clears the ground for post-anthropocentric (Braidotti, 2011: 327) sociology, shifting humans from the central focus of sociological attention and facilitating a posthuman sociology to engage productively with the world beyond the human: with other living things, and with the wider environment of matter and things. By challenging any distinction between the materiality of the physical world and the social constructs of human thoughts and desires, it enables exploration of how each affects the other, and how things other than humans (for instance, a tool, a technology or a building) can be social ‘agents’, making things happen. This flattening of the nature/culture dualism is applicable not only when exploring topics such as environmental change, technology or science, but also to re-think the part that the non-human and non-animate, matter and meaning play in social production more generally (Karakayali, 2015), for instance in education (Alldred and Fox, 2017) or public health (Fox and Alldred, 2016).

Second, a sociological imagination in which there are no structures, no systems and no mechanisms at work means focusing much more intently upon ‘events’: the endless cascade of events comprising the material effects of both nature and culture that together produce the world and human history (Sotiris, 2016: 303). Indeed, this move dramatically simplifies the project of sociological explanation. In place of a search for elusive structures or mechanisms, the agenda for social inquiry re-focuses upon the micropolitics of the world of events (Latour, 2005: 65–6); power or resistance are explained in terms of assemblage micropolitics and the capacities produced in bodies, things and social formations. Post-structuralism questioned the idea of a coercive ‘top-down’ power, arguing instead that power in the contemporary world is disciplinary or governmental, productive of subjectivities and dispositions (Foucault, 1979; Rose, 1999). The perspective applied in this article goes further, to establish a micropolitics of power and resistance amenable to empirical exploration in terms of the affective fluxes within events. The terms ‘power’ and ‘resistance’ may offer the impression of much more concerted social processes, whereas at the level of an event the flux of forces in assemblages can often shift the capacities of bodies or collections of bodies from moment to moment.

Third, feminists, postcolonial scholars, queer theorists and other socially and politically engaged scholars have suggested that the radical monism of materialism and posthumanism ensures that social theory is embedded and embodied in the materiality of life and struggle (Braidotti, 2011: 128; Grosz, 1994: 164), and is hence a means both to research the social world and change it for the better. Though post-structuralism challenged top-down, determinist theories of power and social structure, a focus upon textuality, discourses and systems of thought in these approaches tended to create distance between theory and practice, and gave the sense that radical, interventionist critiques of inequities and oppressions were little more than further constructions of the social world (Coole and Frost, 2010: 25; Edwards, 2010: 282). At the same time, as we saw in our analysis of power and resistance earlier, monist sociology offers a radical critique of essentialism, placing in question ontologies that posit entities with pre-existing attributes (such as abilities, genders) or a fixed stable reality in which power is asserted by one party and resistance mounted by another. Replacing sociological dualisms with multiplicities acknowledges the emergent character of the world and all the possibilities this implies (Barad, 2001: 77; Braidotti, 2013: 60).

Our intention in this article has been to examine the impact of a flattened ontology of the social world for sociology, rather than specifically to advocate materialist or posthuman perspectives. We retain reservations about both of the ‘manifestos’ for non-binary sociology that we considered earlier. For us, DeLanda places too much emphasis upon the stability and continuities of social assemblages, whether person-to-person interaction or city or nation assemblages. This, we would argue, is due largely to his rigid stratification of assemblages into a hierarchy of levels, which emphasises social institutions rather than events, and does not fully acknowledge the interactions between micro and macro that bring both fluidity and stability to assemblages. We find in actor-network theory, even in Latour’s latest formulations, a residual essentialism associated with the entities (whether a body or a physical object such as a laboratory or a technology) identified in ANT’s empirical studies that does not fully acknowledge the exteriority of their relations and capacities (cf. Cudworth and Hobden, 2015: 138). To inform the sociological imagination, a monistic sociology might usefully draw not only upon these scholars, but also on Braidotti’s (2006, 2013) analysis of anthropocentrism and posthumanism, Bennett’s (2010) vitalist ecology, and the Spinozist theorising of affect in Deleuze and Guattari (1988: 260) and others.

## Notes

1. The ‘new’ materialisms encompass disparate perspectives, including actor-network theory, artificial intelligence, biophilosophy, evolutionary theory, feminism, neuroscience, non-representational theory, posthumanism, queer theory, quantum physics and Spinozist monism. For more on these, see Ansell Pearson (1999); Fox and Alldred (2015, 2017); Coole and Frost (2010); Thrift (2004).

2. Paradoxically, the monism of the new materialisms is not a move to universalism or a unitary perspective upon the social or upon subjectivity; rather, a rejection of dualisms opens up a multiplicity and diversity that exceeds and overwhelms the dichotomies they replace (Braidotti, 2011: 211; van der Tuin and Dolphijn, 2010: 158). One equals many in this ontology – as Deleuze (2001: 95) has it: ‘the only enemy is two’. Multiplicity is acknowledged variously throughout new materialist thought: in Deleuzo-Guattarian notions of rhizome (Deleuze and Guattari, 1988: 30), nomadology (1988: 380–1) and becoming (1988: 239); in Barad’s diffractive methodology (2007: 90); in Mol’s (2002) body-multiple; and in Braidotti’s (2011: 211) nomadic subject.

3. In practice, this ‘pure’ capitalist transaction is trammelled by further affects that inhibit its completion, such as sales taxes or tariffs, regulations on safety of goods or consumer protection, international trade rules, geographical barriers, or cultural values such as those that have until recently kept whole areas of social life such as education, health care and religion off-limits to markets.

4. This Nietzschean understanding of the immanence of power is common to the work of both Deleuze and Foucault (Patton, 2000: 49–52, 55).
